# Concomitant Active Tuberculosis Prolongs Survival in Non-Small Cell Lung Cancer: A Study in a Tuberculosis-Endemic Country

**DOI:** 10.1371/journal.pone.0033226

**Published:** 2012-03-16

**Authors:** Chih-Hsi Kuo, Chun-Yu Lo, Fu-Tsai Chung, Kang-Yun Lee, Shu-Min Lin, Chun-Hua Wang, Chih-Chen Heh, Hao-Cheng Chen, Han-Pin Kuo

**Affiliations:** 1 Department of Thoracic Medicine, Chang Gung Memorial Hospital, Taipei, Taiwan; 2 Department of Thoracic Medicine, St. Paul’s Hospital, Taoyuan, Taiwan; 3 Department of Thoracic Medicine, Taipei Medical University, Shuang-Ho Hospital, Taipei, Taiwan; National Taiwan University Hospital, Taiwan

## Abstract

**Background:**

Adjuvant tumor cell vaccine with chemotherapy against non-small cell lung cancer (NSCLC) shows limited clinical response. Whether it provokes effective cellular immunity in tumor microenvironment is questionable. Concomitant active tuberculosis in NSCLC (TBLC) resembles locoregional immunotherapy of tumor cell vaccine; thus, maximally enriches effective anti-tumor immunity. This study compares the survival and immunological cell profile in TBLC over NSCLC alone.

**Methods:**

Retrospective review of NSCLC patients within 1-year-period of 2007 and follow-up till 2010.

**Results:**

A total 276 NSCLC patients were included. The median survival of TBLC is longer than those of NSCLC alone (11.6 vs. 8.8 month, *p*<0.01). Active tuberculosis is an independent predictor of better survival with HR of 0.68 (95% CI, 0.48∼0.97). Squamous cell carcinoma (SCC) (55.8 vs. 31.7%, *p*<0.01) is a significant risk factor for NSCLC with active TB. The median survival of SCC with active tuberculosis is significantly longer than adenocarcinoma or undetermined NSCLC with TB (14.2 vs. 6.6 and 2.8 months, *p*<0.05). Active tuberculosis in SCC increases the expression of CD3 (46.4±24.8 vs. 24.0±16.0, *p*<0.05), CXCR3 (35.1±16.4 vs. 19.2±13.3, *p*<0.01) and IP-10 (63.5±21.9 vs. 35.5±21.0, *p*<0.01), while expression of FOXP3 is decreased (3.5±0.5 vs. 13.3±3.7 *p*<0.05, *p*<0.05). Survival of SCC with high expression of CD3 (12.1 vs. 3.6 month, p<0.05) and CXCR3 (12.1 vs. 4.4 month, p<0.05) is longer than that with low expression.

**Conclusions:**

Active tuberculosis in NSCLC shows better survival outcome. The effective T lymphocyte infiltration in tumor possibly underlies the mechanism. Locoregional immunotherapy of tumor cell vaccine may deserve further researches.

## Introduction

Non-small cell lung cancer (NSCLC) remains the leading cause of cancer death worldwide[1]. Chemotherapy alone has been the standard care in advanced disease, albeit the effect is suboptimal[2], [3]. Adjuvant immunotherapy in combination with chemotherapy has been reported to be an alternative, which improves quality of life without the increase of side effect. Hence, understanding the host immune responses to NSCLC is essential to tailor the use of immunotherapeutic strategies.

The “immune surveillance theory“ holds that all tumor cells express antigenic markers capable of eliciting immune responses which prevent the outgrowth of malignant cells[4]. However, cellular immunity is usually ineffective because neoplasms have mechanism to evade the host immune response [5], [6]. Firstly, tumor antigens are usually poorly immunogenic because they are perceived by the immune system as “self” and “alter self” antigens. Secondly, tumor cells may induce functional suppression of T cell, and conferring resistance to T cells-induced apoptosis. In contrast, increased T cell infiltration in tumor site has been reported to be positively associated with survival outcome in NSCLC [5], [6].

Therefore, correction of cancer-related host immune dysregulation accounts for an appealing strategy of anti-cancer treatment. Purified protein derivative of *M. tuberculosis* (PPD) enhances the production of inducible protein-10 (IP-10), the ligand of CXCR3, in human lungs [7], [8] to recruit activated T cells. Increased CXCR3 expression in tumor nests is associated with prolonged survival and more inflammatory cell infiltration in patients with non-small cell lung cancer (NSCLC) [9]. Tumor cell vaccine, such as tuberculosis (TB) Bacillus Calmette-Guérin (BCG) and heat-killed *Mycobacterium vaccae* suspension (SRL172), have been used as non-specific immunostimulator against several types of human cancers with variable clinical response[10], [11]. For advanced NSCLC, combination of SRL172 with chemotherapy showed limited improvement of survival but improvement in quality of life [12].

**Table 1:** Baseline patients characteristics (N = 276).

Variables	No, (%)
Age, yr, mean±SD	67.0±9.2
Male sex	192 (69.6)
**Performance status**	
ECOG ≤ 1	148 (53.6)
ECOG ≥ 2	128 (46.4)
**Stage**	
Stage IIIA	11 (4.0)
Stage IIIB	98 (35.5)
Stage IV	167 (60.5)
**Pathological subtype**	
Adenocarcinoma	129 (46.7)
Squamous cell carcinoma	100 (36.2)
NSCLC, undetermined	47 (17.1)
**Smoking status**	
Smoker or ex-smoker	172 (62.3)
Non- smoker	104 (37.7)
**Treatment**	
Chemotherapy	171 (62.0)
Radiotherapy	36 (13.0)
Target therapy	216 (78.3)
BSC alone	72 (26.1)
**Tuberculosis**	
With TB	52 (18.8)
Without TB	224 (81.2)

NSCLC: non-small cell lung cancer.

BSC: best supportive care, TB: tuberculosis.

**Figure 1:**
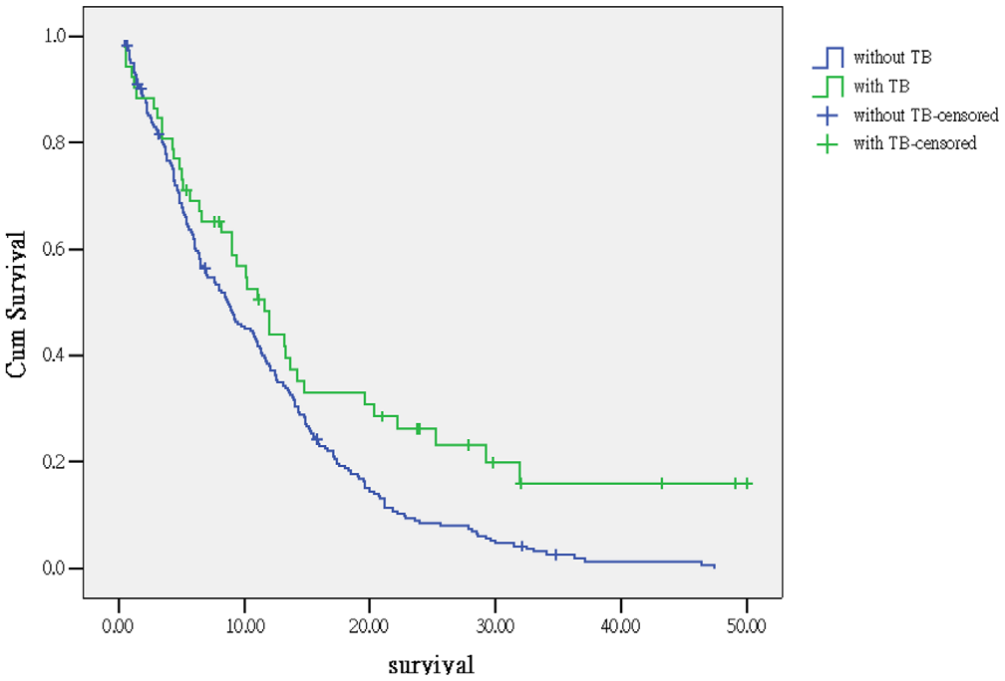
Kaplan-Meier survival curve of lung cancer patients with and without active tuberculosis (log rank test, *p*<0.01) . Green line: patients with TB. Blue line: patients without TB.

The wide variation of efficacy of tumor cell vaccine raised a question of whether and to what extent is the T cell immunity provoked by systemic injection of tumor cell vaccine enriches in the tumor microenvironment to potentiate effective immunity against tumor[13], [14] In a tuberculosis endemic country, it is possible to approach it[15] by looking at the immunological cell profile in tumor microenvironment when NSCLC has superimposed active TB happening in close vicinity[15]. Furthermore, this clinical scenario resembles the locoregional immunotherapy of tumor cell vaccine for NSCLC, and suggests the development of a constant and maximally enriched T cell immunity nearby tumors.

**Figure 2:**
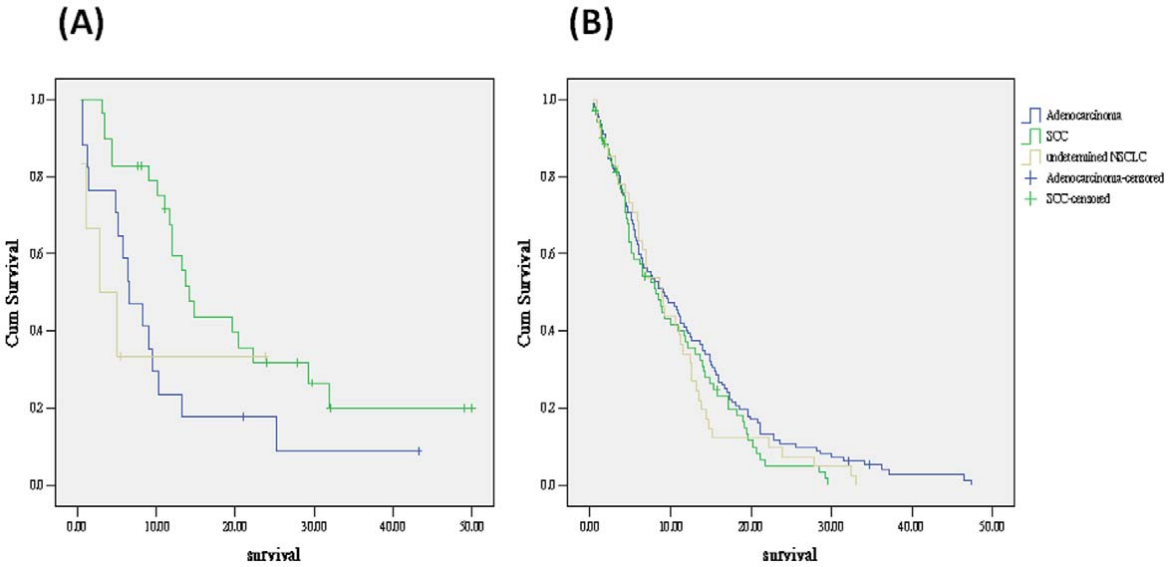
Kaplan-Meier survival curve of (A) NSCLC with active TB grouped by histology (log rank test, *p*<0.05). (B) NSCLC without TB grouped by histology (log rank test, *p* = 0.27). Green line: SCC, Blue line: adenocarcinoma, Yellow line: undetermined NSCLC. SCC: squamous cell carcinoma NSCLC: non-small cell lung cancer

**Figure 3:**
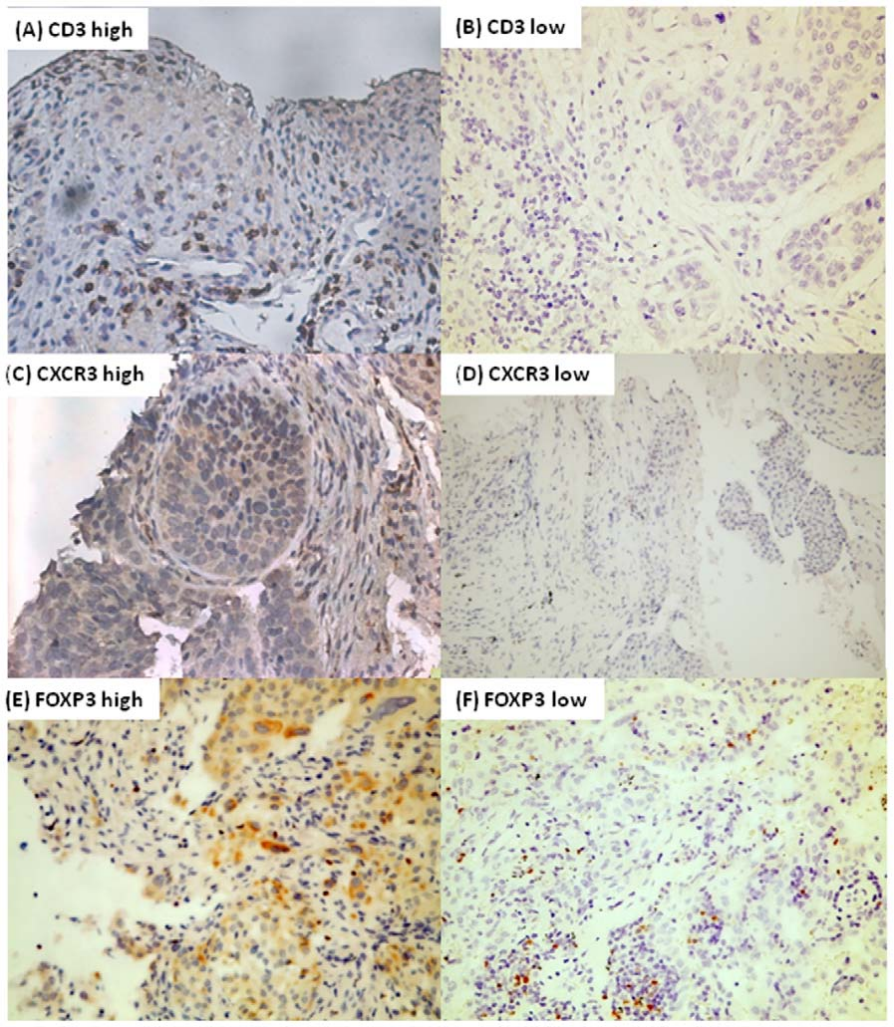
Representative image of immunohistochmical stain demonstrating immunological markers with high and low expression respectively for CD3 (A) (B), CXCR3 (C) (D), and FOXP3 (E) (F) . Magnification×400.

This study primarily aims to compare the outcome in NSCLC patients with concomitant active TB over NSCLC alone patients, in terms of overall survival. Furthermore, the expression of immunological cell profile adjacent to tumors in either group is also studied.

## Materials and Methods

### Study population

We performed a retrospective review of active tuberculosis in advanced NSCLC patients in Chang Gung Memorial Hospital (a tertiary referral medical center) during the 1-year period of 2007 and followed up their clinical outcomes till 2010. Informed consents were obtained for all stored samples used for this study. Determination of active TB infection started from the review of positive acid-fast smear from sputum, bronchial wash or broncho-alveolar lavage fluid in advanced NSCLC patients, followed by the specific focus on those with positive TB growth to assure the existence of viable mycobacteria. Hence, active TB infection qualified both positive acid-fast smear and positive culture for TB. Subsequently, the chest X-ray or CT scan were reviewed to include patients presenting same lobar distribution of TB and cancerous lesions.

**Table 2:** Predicting factors of survival outcome by univariate and multivariate analysis.

	Median	Univariate		Multivariate	
Variables (No.)	survival	HR (95% CI)	*p*-value	HR (95% CI)	*p*-value
	(month)				
**Performance status**					
ECOG ≤ 1 (148)	13.7				
ECOG ≥ 2 (128)	5.9	0.43 (0.33∼0.55)	<0.01	0.41 (0.29∼0.57)	<0.01
**Tuberculosis**					
With TB (52)	11.6				
Without TB (224)	8.8	0.61 (0.43∼0.86)	<0.01	0.68 (0.48∼0.97)	<0.05
**Pathological subtype**					
Squamous cell carcinoma(100)	11.1				
Adenocarcinoma (129)	8.4	0.73 (0.51∼1.05)	0.10		
NSCLC, undetermined (47)	8.7	0.83 (0.59∼1.17)	0.29		
**Gender**					
Female (84)	10.7				
Male (192)	8.7	0.83 (0.63∼1.08)	0.16	0.63 (0.48∼0.83)	<0.05
**Stage**					
Stage III (109)	12.5				
Stage IV (167)	8.2	0.64 (0.50∼0.83)	<0.01	0.62 (0.47∼0.80)	<0.01
**Smoking status**					
Non-smoker (104)	9.4				
Smoker or ex-smoke (172)	9.0	0.91 (0.71∼1.17)	0.47		
**Treatment**					
With treatment (204)	11.6				
BSC alone (72)	6.6	0.54 (0.41∼0.72)	<0.01	0.90 (0.63∼1.30)	0.58

NSCLC: non-small cell lung cancer, HR: hazard ratio, BSC: best supportive care, TB: tuberculosis.

### Staging and treatment of advanced NSCLC

The scheme of 2009 AJCC 7^th^ edition for TNM staging was used for study patients. Platinum-based chemotherapy was the standard care for stage IV and stage IIIB patients. Stage IIIA patients received concurrent chemoradiotherapy (CCRT) or platinum-based neoadjuvant chemotherapy, and surgical resection was considered for whom have been downstaged successfully. Performance status is measured by ECOG scoring system runs from 0 to 5 [16]. Patients with an ECOG performance status more than two were treated with single-agent chemotherapy or best supportive care alone decided by in charge physician.

**Table 3:** Analysis of clinical factors associated with active tuberculosis in NSCLC patients.

	With TB (%)	Without TB (%)	
Variables	N = 52	N = 224	*p-value*
Age, yr, mean±SD	67.4±9.6	66.7±10.2	0.95
Male sex	82.7	66.5	<0.05
**Performance status**			
ECOG ≤ 1	53.8	53.6	1.00
ECOG ≥ 2	46.2	46.4	
**Stage**			
Stage III	44.2	38.4	0.44
Stage IV	55.8	61.6	
**Pathological subtype**			
Adenocarcinoma	32.7	50.0	<0.01*
Squamous cell carcinoma**	55.8	31.7	
NSCLC, undetermined	11.5	18.3	
**Smoking status**			
Smoker or ex-smoker	78.8	58.5	<0.01
Non- smoker	21.2	41.5	
**Treatment**			
Chemotherapy	61.5	62.1	
Radiotherapy	11.5	13.4	
Target therapy	71.1	79.9	
BSC alone	25.0	26.3	0.97

*comparison of squamous vs. non-squamous cell carcinoma

**(ORs 2.09; 95% CI, 1.06∼4.14, *p*< 0.05) by multivariate logistic regression test

NSCLC: non-small cell lung cancer, BSC: best supportive care, TB: tuberculosis.

### Treatment for tuberculosis

Patients with concomitant active tuberculosis received standard treatment with two months isoniazide, rifampicin, pyrazinamide and ethambutol combination, following by four months combination with isoniazide, rifampicin and ethambutol. For patients whose treatment of NSCLC overlapped with that of TB; the former was postponed at least one month after the start of anti-tuberculosis therapy. The appropriate timing to resume the NSCLC treatment was decided by in charge physician based on patient’s performance status.

### Immunohistochemical (IHC) staining

Paraffin-embedded specimens were retrieved. Rabbit anti-human antibodies to CD3 (Dako A/S, Glostrup, Denmark), CD4 (NeoMarkers, Thermo Fisher Scientific, Fremont, CA, U.S.A.), CD8 (BioCare Medical, Concord, CA, U.S.A.), CD56 (Dako A/S, Glostrup, Denmark), CD 68 (Dako A/S, Glostrup, Denmark), iNOS (Santa Cruz Biotechnology, CA, U.S.A.), CXCR3 (BD Biosciences Pharmingen, NJ, USA), IP-10 (Santa Cruz Biotechnology, CA, U.S.A.), CD25 (Novocastra, Newcastle upon Tyne, UK) and FOXP3 (ab20034; Abcam, Cambridge, UK) were obtained. The staining intensity in tumor cells was assessed in comparison to normal bronchial epithelium as an internal positive control. Staining specificity was evaluated by negative controls in which the primary antibody had taken the place of normal mouse (for CD3, CD4, CD56, CD68, CD25 and FOXP3) and normal rabbit (for CD8, iNOS, CXCR3 and IP-10) non-immune IgG. The IHC slides were assessed under a light-microscope at X400 magnification. All nucleated cells in tumor islet cells and stroma cells (including tumoral, structural and infiltrative inflammatory cells) were counted by reader blinded for survival outcome information. The percentage of cell marker-positive nucleated cells of total nucleated cells in selective tumor islets and stromal areas was calculated and expressed as cells/100 nucleated cells.

**Table 4:** Immunohistochemical marker indices of squamous cell carcinoma with or without active tuberculosis.

Marker indices	With TB	Without TB	
%, ( mean±SD)	N = 13	N = 18	*p*-value
**CD3**	46.4±24.8	24.0±16.0	<0.05
**CD4**	6.4±12.2	2.0±2.0	0.29
**CD8**	10.1±10.1	10.8±6.9	0.48
**CD68**	19.2±12.4	16.0±9.3	0.80
**iNOS**	53.4±31.9	39.5±35.5	0.31
**CD56**	6.3±3.5	8.4±7.7	0.86
**CXCR3**	35.1±16.4	19.2±13.3	<0.01
**IP-10**	63.5±21.9	35.5±21.0	<0.01
**CD25**	31.6±2.9	28.9±2.3	0.47
**FOXP3**	3.5±0.5	13.3±3.7	<0.05

TB: tuberculosis.

### Statistical analysis

All quantitative data were expressed as mean values and standard deviations. Non-parametric approaches were used since most data were not normally distributed. The Mann–Whitney test was used to compare numeric variables between two groups, and the χ^2^ test was used for categorical variables. Univariate analyses of survival used the log-rank test to examine effects of baseline clinical factors. Variables with a value of *p*<0.1 was made to enter a multivariate analysis with Cox’s regression model for identifying independent predictor of survival. All analyses were conducted using the Statistical Package for the Social Sciences (SPSS, v. 13.0; SPSS Inc, Chicago, IL) and GraphPad Prism software package (v. 5; GraphPad Prism Software Inc, San Diego, CA). All comparisons with a *p*-value less than 0.05 were considered statistically significant.

## Results

### Patients characteristics

A total 276 advanced NSCLC patients were included, with 11 (4.0%) stage IIIA, 98 (35.5%) stage IIIB and 167 (60.5%) stage IV. One hundred fifteen patients received first-line platinum-based doublet, with gemcitabine in 63 (54.8%), docetaxel in 18 (15.7%), paclitaxol in 13 (11.3%), vinorelbine in 16 (13.9%) and pemetrexed in 5 (4.3%). Thirty patients received first-line single agent chemotherapy, with gemcitabine in 16 (53.3%), docetaxel in 8 (26.7%), paclitaxol in 2 (6.7%), vinorelbine in 4 (13.3%). Fifty-nine and seventy-two patients were respectively placed on first-line target therapy and best supportive care alone. In active TB group, nine (17.3%) had suspicious old pulmonary TB lesions based on image studies and four (7.7%) had documented history of previous anti-TB therapy. Eight (15.4%) out of fifty-two showed biopsy-proven caseous granulomatous inflammation on histology. Table 1 shows the baseline characteristics of all patients.

**Table 5:** Survival outcome analysis of high or low immunohistochemical marker indices divided by median in squamous cell carcinoma.

Markers	Median survival (month)	*p*-value
**CD3**		
low	3.6	
high	12.1	<0.05
**CD4**		
low	7.6	
high	8.5	0.80
**FOXP3**		
low	13.0	
high	8.1	0.49
**CXCR3**		
low	4.4	
high	12.1	<0.05
IP-10		
low	4.5	
high	9.9	0.37

### Active Tuberculosis as an independent predictor of survival outcome

Predictors of survival outcome are shown in Table 2. In univariate analysis, better performance status, TB infection, stage III of NSCLC and receiving treatment are significant predictors for survival outcome with HR of 0.43 (95% CI, 0.33∼0.52), 0.61 (95% CI, 0.43∼0.86), 0.62 (95% CI, 0.48∼0.79) and 0.54 (95% CI, 0.41∼0.72), respectively. In multivariate analysis, active TB remains one of the independent predictors of survival outcome with HR of 0.68 (95% CI, 0.48∼0.97). Figure 1 shows the survival curve of NSCLC patients with or without active TB. The median survival of concomitant active TB is longer than those without TB (11.6 vs. 8.8 month, *p*<0.01).

### Squamous cell carcinoma is a risk factor for concomitant active tuberculosis

Table 3 shows the clinical factors of NSCLC patients with or without active tuberculosis. The risk of squamous cell carcinoma (55.8 vs. 31.7%), male gender (82.7 vs. 66.5%) and smoker (78.8 vs. 58.5%) are significantly higher for NSCLC with active TB. Multivariate analysis shows that squamous cell carcinoma (ORs 2.09; 95% CI, 1.06∼4.14, *p*<0.05) is the single independent risk factor.

### Concomitant active tuberculosis shows better survival outcome in squamous cell carcinoma patients

Comparison of survival outcome stratified by tuberculosis infection and histology are shown in Figure 2A (with TB) and Figure 2B (without TB). In NSCLC with concomitant active TB, the median survival is significantly longer in squamous cell carcinoma than adenocarcinoma and undetermined NSCLC (14.2 vs. 6.6 and 2.8 months, *p*<0.05). For patient without TB, survival is not significantly different between three histology groups (8.2, 9.1 and 8.9 month, *p* = 0.27).

### Increases in CD3, CXCR3 and IP-10 expression in squamous cell carcinoma with concomitant active tuberculosis

Immunological cell and chemokine profile adjacent to squamous cell carcinoma was studied by immunohistochemical stain to assess the markers representative of T lymphocyte (CD3, CD4, CD8 and CD56), regulatory T cell (CD25, FOXP3) macrophage (CD68, iNOS) and related chemokine or chemokine receptor (IP-10 and CXCR3). The indices presented by percentage of each marker adjacent to squamous cell carcinoma is shown in Table 4. Compared to squamous cell carcinoma alone, concomitant active TB significantly increases the expression of CD3 (46.4±24.8 vs. 24.0±16.0, *p*<0.05), CXCR3 (35.1±16.4 vs. 19.2±13.3, *p*<0.01) and IP-10 (63.5±21.9 vs. 35.5±21.0, *p*<0.01), while expression of FOXP3 is significantly decreased (3.5±0.5 vs. 13.3±3.7 *p*<0.05). For adenocarcinoma with concomitant active TB, significantly higher expression of CD3 and CXCR3 are also noted. (Table S1)

### Increases in CD3, CXCR3 expression is associated with better survival outcome in squamous cell carcinoma patients

The survival outcome analysis of high or low immunohistochemical marker indices divided by median in squamous cell carcinoma is shown in Table 5. Tumor with high expression of CD3 (12.1 vs. 3.6 month, p<0.05) and CXCR3 (12.1 vs. 4.4 month, p<0.05) shows better median survival than those with low expression, while other markers are not associated with survival outcome. Figure 3 representatively demonstrates the images showing high and low expression of CD3, CXCR3 and FOXP3. For adenocarcinoma, level of expression for each inflammatory marker is not associated with survival outcome. (Table S2)

## Discussion

The present study has demonstrated that NSCLC with active TB is more frequently found in squamous cell carcinoma. With concomitant active TB, NSCLC patients show a better survival outcome; especially in squamous cell carcinoma, which is associated with increased CD3- and CXCR3-expressing cells within tumor.[17], [18], [19]

A clinicopathological analysis reported by Tamura et al. have shown that mycobacteriosis is often adjacent to preexisting lung cancer. [20] The walls of encapsulated caseous nodules may be invaded by NSCLC, especially squamous cell carcinoma featuring local invasiveness; thereby, leading to tuberculosis reactivation. Our result reveals that active TB infection in NSCLC is an independent predictor of better survival. There is abundant evidence in the earlier literature that remission, regression and even total disappearance of tumors may occur when mycobacterium infection co-exists. [21] However, treatment with tumor cell vaccine preparation from heat-killed *Mycobacterium vaccae* suspension (SRL172) for advanced NSCLC has only shown limited survival benefit.[12] One of the aims of tumor cell vaccine therapy is correcting the imbalance of Th1 and Th2 cell immunity established by cancer cells. [22], [23] These include: induction of T lymphocytes accumulation [24], upregulation of Th1 cytokines, such as interferon-γ [25], and suppression of Th2 immunity [26]. However, whether these immune reactions provoked by intradermal injection of SRL172 are effectively enriched nearby cancer cells has never been confirmed.

By studying NSCLC in close vicinity to active tuberculosis, it resembles locoregional immunotherapy of tumor cell vaccine for lung cancer. The inflammatory profile nearby tumor in this condition is higher than those without TB, in terms of CD3, CXCR3 and IP-10 for squamous cell carcinoma, and CD3 and CXCR3 for adenocarcinoma. Moreover, when analyzing inflammatory burden to survival, it reveals that higher infiltration of CD3- and CXCR3-expressing cells are associated with better survival in squamous cell carcinoma. This result suggests that locoregional immunotherapy possibly enables enrichment of effective anti-tumor immunity nearby tumor, but appears to be important for the clinical outcomes in patients with squamous cell carcinoma.

On the other hand, our results show that CD3 and CXCR3 rather than IP-10 are the markers associated with survival benefit. There are two possible explanations to it. Firstly, IP-10 is not the ligand exclusively required by CXCR3 in order to activate T lymphocyte [8], whereas CXCL9 and CXCL11 could play the similar function. Secondly, the activation of T lymphocyte not only depends on the ligand stimulation, but also on the immunologically suppressive cells recruited and tamed by tumor. The emerging understanding of regulatory T cells (Treg) significantly improves our knowledge of immune system evasion and how it enables tumor progression.[22], [27], [28] Our study also provides evidence that expression of FOXP3; as a marker for Treg, is decreased in squamous cell carcinoma with active TB. Dumitriu et al. has shown that NSCLC promotes the differentiation of Treg through dendritic cell via a TGF-beta 1 dependent mechanism[29]. In light of this, report from MacKinnon et al. revealed that blockade of galectin-3 could possibly inhibit the IL-4/IL-13 driven alternative macrophage activation which is critical in building a tumor friendly extracellular matrix[30]. Therefore, gaining better insight of the immunosuppressive property of tumor microenvironment underlies the responsiveness of tumor cell vaccine treatment. The major limitation of current study is the retrospective property per se; thereby, the samples qualified for immunohistochemical stain are limited due to lack of prospective standardization. However, the trend of survival outcome is similar with the main clinical results. Additionally, the possibility of synergistic effect or interaction between the anti-tuberculosis medication and the chemotherapeutic agents could not be excluded.

In conclusion, this study shows that NSCLC with active TB has better survival outcome. The effective T cell immunity nearby tumor possibly underlies the mechanism. Locoregional treatment of tumor cell vaccine for NSCLC may deserve further researches.

## Supporting Information

Table S1 **Immunohistochemical marker indices of adenocarcinoma with or without active tuberculosis**.
(PDF)

Table S2 **Survival outcome analysis of high or low immunohistochemical marker indices divided by median in adenocarcinoma**.
(PDF)
